# *Lactobacillus acidophilus *induces a slow but more sustained chemokine and cytokine response in naïve foetal enterocytes compared to commensal *Escherichia coli*

**DOI:** 10.1186/1471-2172-11-2

**Published:** 2010-01-19

**Authors:** Louise H Zeuthen, Lisbeth N Fink, Stine B Metzdorff, Matilde B Kristensen, Tine R Licht, Christine Nellemann, Hanne Frøkiær

**Affiliations:** 1 Center for Biological Sequence Analysis, Department of Systems Biology, Technical University of Denmark, 2800 Kgs. Lyngby, Denmark; 2 Department of Basic Sciences and Environment, Faculty of Life Sciences, University of Copenhagen, 1870 Frederiksberg, Denmark; 3 Department of Toxicology and Risk Assessment and Department of Microbiology, National Food Institute, Technical University of Denmark, 2860 Mørkhøj, Denmark

## Abstract

**Background:**

The first exposure to microorganisms at mucosal surfaces is critical for immune maturation and gut health. Facultative anaerobic bacteria are the first to colonise the infant gut, and the impact of these bacteria on intestinal epithelial cells (IEC) may be determinant for how the immune system subsequently tolerates gut bacteria.

**Results:**

To mirror the influence of the very first bacterial stimuli on infant IEC, we isolated IEC from mouse foetuses at gestational day 19 and from germfree neonates. IEC were stimulated with gut-derived bacteria, Gram-negative *Escherichia coli *Nissle and Gram-positive *Lactobacillus acidophilus *NCFM, and expression of genes important for immune regulation was measured together with cytokine production. *E. coli *Nissle and *L. acidophilus *NCFM strongly induced chemokines and cytokines, but with different kinetics, and only *E. coli *Nissle induced down-regulation of Toll-like receptor 4 and up-regulation of Toll-like receptor 2. The sensitivity to stimulation was similar before and after birth in germ-free IEC, although Toll-like receptor 2 expression was higher before birth than immediately after.

**Conclusions:**

In conclusion, IEC isolated before gut colonisation occurs at birth, are highly responsive to stimulation with gut commensals, with *L. acidophilus *NCFM inducing a slower, but more sustained response than *E. coli *Nissle. *E. coli *may induce intestinal tolerance through very rapid up-regulation of chemokine and cytokine genes and down-regulation of Toll-like receptor 4, while regulating also responsiveness to Gram-positive bacteria.

## Background

The human gastrointestinal (GI) tract, the largest surface area of the body in contact with the environment, is lined by a single layer of intestinal epithelial cells (IEC). In adults, the GI tract is colonised by more than 10^14 ^microorganisms comprising more than 500 different phylotypes [1]. The gut microbiota is pivotal for the development and maintenance of intestinal immunological homeostasis. The intestinal epithelium plays key roles in maintaining this immune homeostasis in the gut as an active player in maintaining tolerance to the microbiota and food antigens as well as in pathogen combat.

The GI tract of the foetal baby is sterile, but colonisation starts immediately after birth with bacteria from the mother and the environment and, within a few days, it is colonised by numerous bacterial species. These pioneer bacteria have been shown to modulate gene expression in IEC including genes involved in metabolism, absorption, barrier function and IEC maturation [2]. Colonisation at birth by facultative anaerobes, such as enterobacteria, coliforms, lactobacilli and streptococci, creates a reducing environment during the first week of life enabling colonisation by strict anaerobes including bifidobacteria, bacteroides, clostridia and eubacteria [3]. This microbial colonisation contributes to recruitment of immune cells to the GI tract and may furthermore be a major contributor to establishment of the systemic immune system [4,5]. Thus colonisation in early infancy is crucial in relation to the final composition of the permanent microbiota in adults and also in inducing intestinal and immunological maturation.

IEC sense commensals through expression of pattern recognition receptors (PRRs) recognising conserved microbial structures. The IEC respond by secreting a wide range of chemokines that recruit immune cells to the GI tract, and cytokines that affect the immune cells scattered in the GI tract including DC, macrophages and lymphocytes [6-9]. Due to the heavy bacterial antigen load in the lumen, the expression of PRRs is tightly regulated in IEC. IEC express Toll-like receptor (TLR) 1-9 [10], nucleotide-binding oligomerisation domain (NOD) 1 and NOD2 [11]. However, contradicting data from cell line studies on the expression of TLRs in IEC exist. Several reports demonstrate that IEC are non-responsive towards lipopolysaccharide (LPS) and express no or very low levels of TLR4 [12,13], while other groups have reported the presence of TLR4 [10,14,15]. This discrepancy may be explained by the finding that IEC gain a cross-hyporesponsive phenotype after stimulation with either LPS or lipoteichoic acid due to decreased signalling through TLR2 and TLR4 [10]. Cario et al. elegantly demonstrated that both TLR2 and TLR4 are constitutively expressed apically in an IEC cell line but traffic to cytoplasmic compartments after ligand stimulation [14]. IEC isolated from intestinal tissue express *Tlr2 *and *Tlr4 *mRNA but at low levels both in humans [16] and mice [17]. Knowledge on IEC responses to microbe-associated molecular patterns (MAMPs) is to a large extent based on cell line studies as cell lines are naïve to MAMP stimulation. However, cell lines may not entirely reflect IEC responses at birth.

Besides playing a role in the recruitment and maturation of immune cells in the GI tract, the bacteria colonising the sterile gut probably induce tolerance dependently on TLR-activation [18]. In this respect, the MAMPs present in the first-coming species might be crucial in tolerance development. It was recently demonstrated that, although both foetal and neonatal IEC express the TLR4/MD-2 receptor complex, they differ dramatically in their responsiveness to LPS, and it was suggested that intestinal bacterial colonisation in the newborn is facilitated by postnatal establishment of IEC tolerance towards LPS stimulation [19]. Moreover, IEC help maintaining the specialised intestinal tolerogenic environment through secretion of different mediators, such as thymic stromal lymphopoietin (TSLP) and transforming growth factor (TGF)-β and commensals differentially affect TSLP and TGF-β production [20,21]. Thereby the composition of the microbiota indirectly affects immune cells through effects on IEC.

We hypothesized that the very first bacteria encountered by naive IEC influence the signal molecules released to the gut environment, and that Gram-positive (G^+^) commensals prime IEC differently from Gram-negative (G^-^) commensals and LPS. Moreover, the developmental state of the IEC may play a role in their responsiveness, and this study is the first to compare foetal and neonatal germfree murine IEC responsiveness to G^+ ^(*Lactobacillus acidophilus*) and G^- ^(*Escherichia coli*) commensals *in vitro*. We present indices that the type of bacterial stimulus indeed affects gene expression in naïve primary IEC, thus suggesting an important role of the first postnatal bacteria for immune cell recruitment and tolerance induction in the GI tract.

## Results

### *L. acidophilus *and *E. coli *strongly induce chemokine gene expression in foetal primary epithelial cells in vitro

**Figure 1:**
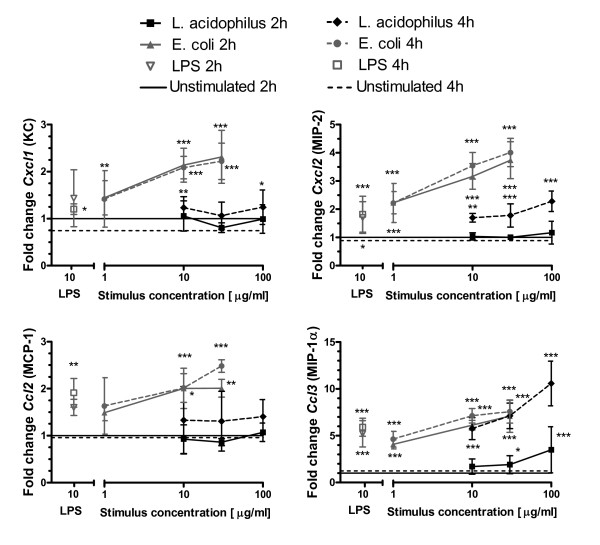
***L. acidophilus *and *E. coli *strongly induce chemokine expression in foetal primary epithelial cells in vitro**. Epithelial cells from foetuses at Day-1 were stimulated for 2 h and 4 h with LPS (10 μg/ml), *L. acidophilus *NCFM (10, 30 and 100 μg/ml) or *E. coli *Nissle (1, 10, 30 μg/ml). Gene expression was measured by RT-PCR. Symbols indicate mean fold increase and SD of 4 independent experiments with cells pooled from 6-10 pups. ***p < 0.001, **p < 0.01, *p < 0.05 compared to "unstimulated cells". Data were normalised to *Actb *and then to the average of "unstimulated cells 2 h" from the 4 experiments, which was defined to 1.

In the first days of life, recruitment of immune cells to the gut is probably one of the most important aspects of gut immune maturation. In this respect, IEC play a pivotal role by secreting chemokines attracting specific immune cells. We speculated that the composition of the gut microbiota affects this maturation process by affecting the chemokine expression in IEC, and therefore studied how expression of a set of chemokines in foetal near-term IEC was affected by *in vitro *bacterial stimulation with two gut-derived commensals (Figure 1). As representatives of gut G^+ ^and G^- ^commensals we chose *E. coli *Nissle and *L. acidophilus *NCFM [22] as these strains in earlier studies were found to be potent stimulators of epithelial cell lines [21]. *E. coli *was most potent in up-regulating *Cxcl1*, *Cxcl2*, *Ccl2 *and *Ccl3 *encoding keratinocyte-derived chemokine (KC), macrophage-inflammatory protein (MIP)-2, monocyte chemoattractant protein (MCP)-1 and MIP-1α respectively. Generally, chemokine expression induced by *E. coli *did not increase from 2 to 4 h, whereas induction of *Ccl3 *(encoding MIP-1α ) by *L. acidophilus *reached transcription levels induced by *E. coli *only at 4 h.

### *L. acidophilus *and *E. coli *up-regulate gene expression of pro-inflammatory and regulatory cytokines in foetal epithelial cells in vitro

**Figure 2:**
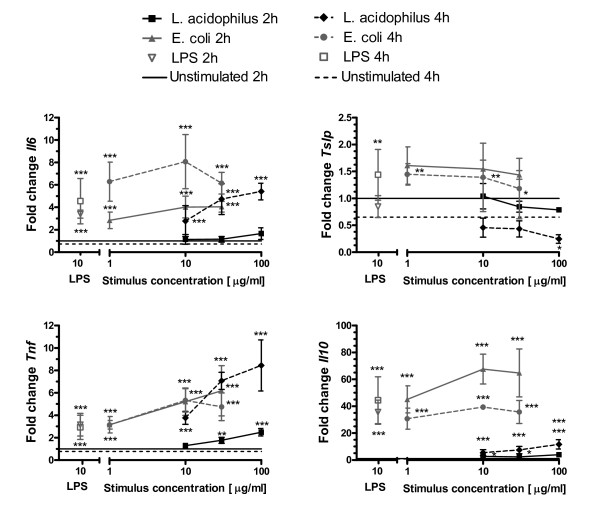
***L. acidophilus *and *E. coli *up-regulate expression of pro-inflammatory and regulatory cytokines in foetal epithelial cells in vitro**. Epithelial cells from foetuses at Day-1 were stimulated for 2 h and 4 h with LPS (10 μg/ml), *L. acidophilus *NCFM (10, 30 and 100 μg/ml) or *E. coli *Nissle (1, 10, 30 μg/ml). Gene expression was measured by RT-PCR. Symbols indicate mean fold increase and SD of 4 independent experiments with cells pooled from 6-10 pups. ***p < 0.001, **p < 0.01, *p < 0.05 compared to "unstimulated cells". Data were normalised to *Actb *and then to the average of "unstimulated cells 2 h" from the 4 experiments, which was defined to 1.

As opposed to chemokine production, cytokine secretion by primary IEC upon bacterial stimulation via their PRRs remains poorly described. Hence we evaluated how *L. acidophilus *and *E. coli *modulate the cytokine environment upon engagement of PRRs *in vitro *in foetal IEC (Figure 2). *L. acidophilus *and *E. coli *induced expression of interleukin (*Il*)*6*, *Il10 *and tumour necrosis factor (*Tnf*) in a dose-dependent manner. *E. coli *and LPS induced *Il6*, *Il10 *and *Tnf *faster than *L. acidophilus*. After 4 h of stimulation, the expression of *Il6 *and *Tnf *induced by *L. acidophilus *reached levels induced by *E. coli*. Induction of *Il10 *by *E. coli *peaked at 2 h while *Il10 *induction by *L. acidophilus *was strongest at 4 h. These differences might imply differences in the kinetics of the two distinct PRR signalling pathways, with signalling through TLR4 being faster than signalling through TLR2. TGF-β and TSLP are known to be secreted by IEC and to induce a tolerogenic DC phenotype [21], hence we also looked at transcription of genes encoding TSLP and TGF-β1. Expression of *Tgfb1 *was not significantly changed upon *in vitro *stimulation with either bacteria (data not shown), while low concentrations of *E. coli *modestly up-regulated expression of *Tslp *and the highest concentration of *L. acidophilus *down-regulated expression of *Tslp *at 4 h.

### Foetal epithelial cells produce cytokines upon in vitro stimulation with Gram-positive and Gram-negative commensals with different kinetics

**Figure 3:**
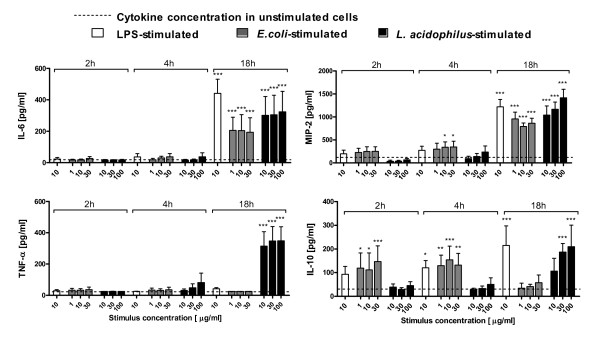
**Foetal epithelial cells produce cytokines upon in vitro stimulation with G^+ ^and G^- ^commensals with different kinetics**. Cytokine and chemokine production in epithelial cells from foetuses at Day-1 after 2 h, 4 h and 18 h stimulation with LPS (10 μg/ml), *L. acidophilus *NCFM (10, 30 and 100 μg/ml) or *E. coli *Nissle (1, 10, 30 μg/ml) measured by ELISA. Symbols indicate mean and SD of 4 independent experiments with cells pooled from 6-10 pups. ***p < 0.001, **p < 0.01, *p < 0.05 compared to "unstimulated cells" represented by dotted lines.

As cytokine production by IEC has been reported to be low [17], we wished to validate the high increases in expression of *Il6*, *Tnf *and *Il10 *by measuring protein secretion from *in vitro *stimulated foetal IEC by enzyme-linked immunosorbent assay (ELISA) at 2 h, 4 h and 18 h of culture (Figure 3). We also measured MIP-2, known to be secreted by IEC [19]. Production of the four proteins correlated well with the transcription levels shown in Figure 1 and 2, as expression of all genes in IEC stimulated with *L. acidophilus *was increasing from 2 h to 4 h, and protein concentrations after 18 h culture were highest for these cells. For *E. coli*-stimulated cells, gene expression was increasing (*Il6*), maintained (*Tnf *and *Cxcl2*) and decreasing (*Il10*) over time and, accordingly, protein levels after 18 h were higher, unchanged, slightly higher and lower for IL-6, TNF-α , MIP-2 and IL-10, respectively, than at the early time points. The chemokine MIP-2 was produced in the highest amounts, but also significant amounts of the three cytokines were produced. The difference between the bacteria again points towards a later induction of certain immunological markers by *L. acidophilus *compared to *E. coli*. LPS induced a higher IL-10 production than *E. coli *after 18 h, perhaps reflecting that LPS-induced expression of *Il10 *was not decreasing from 2 h to 4 h as was *E. coli*-induced *Il10 *expression.

### *E. coli *is more potent than *L. acidophilus *in up-regulating *Tlr2*, *Nfkb1*, and *Nfkb2 *gene expression and in down-regulating expression of *Tlr4 *and *Clec7a *genes in foetal epithelial cells

**Figure 4:**
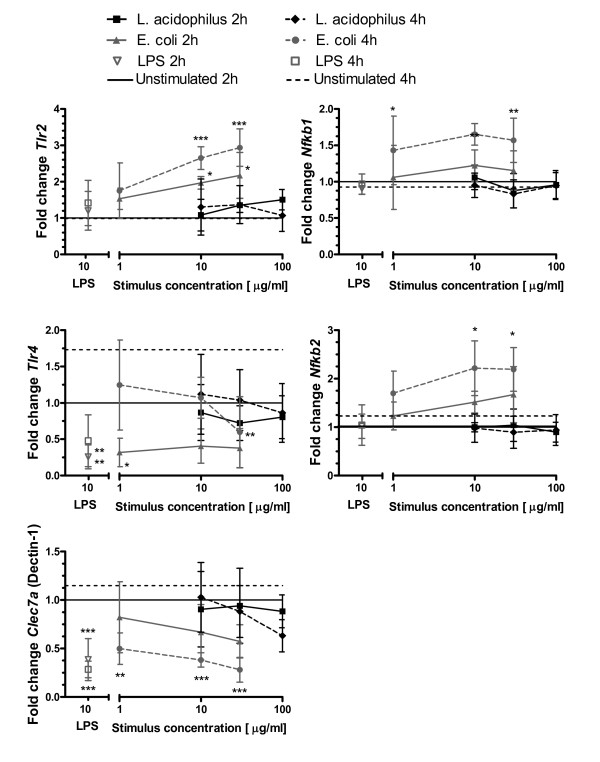
***E. coli *is more potent than *L. acidophilus *in up-regulating Tlr2, Nfkb1, and Nfkb2 expression and in down-regulating expression of Tlr4 and Clec7a in foetal epithelial cells**. Epithelial cells from foetuses at Day-1 were stimulated for 2 h and 4 h with LPS (10 μg/ml), *L. acidophilus *NCFM (10, 30 and 100 μg/ml) or *E. coli *Nissle (1, 10, 30 μg/ml). Gene expression was measured by RT-PCR. Symbols indicate mean and SD of 4 independent experiments with cells pooled from 6-10 pups. ***p < 0.001, **p < 0.01, *p < 0.05 compared to "unstimulated cells". Data were normalised to *Actb *and then to the average of "unstimulated cells 2 h" from the 4 experiments, which was defined to 1.

When IEC sense bacteria, the first and primary interaction is between PRRs and their bacterial ligands. Signalling through the different cascades downstream of PRRs (MyD88 pathway or TIR-domain-containing adapter-inducing interferon-β (TRIF) pathway) then activates transcription of effector genes including genes encoding cytokines and chemokines. Hence, we evaluated the expression of genes encoding TLR2, TLR4, Dectin-1 and MD-2 in near-term foetal IEC after *in vitro *stimulation with *E. coli *Nissle and *L. acidophilus *NCFM. As depicted in Figure 4, *E. coli *(and pure LPS), even at a low concentration (1 g/ml), down-regulated expression of *Tlr4 *significantly. On the contrary, *Tlr2 *expression was strongly enhanced by *E. coli *with strongest effects after 4 h stimulation. Although their transcripts were detected in IEC, no changes in expression of the genes encoding MD-2 (Ly96), IRAK1, IKKβ or Tollip, all recognised to be important regulators of TLR4 signalling were observed (data not shown). Dectin-1 is a PRR known to recognise fungal β-1,3 and β-1,6 linked glucans, which in a Ca^2+ ^independent manner enhances phagocytosis [23]. Dectin-1 is expressed by DC, monocytes, neutrophils, macrophages and in Caco2 IEC [24], but has not previously been studied in primary IEC. As shown in Figure 4, *Clec7a *encoding Dectin-1 was significantly down-regulated after 4 h stimulation with both LPS and *E. coli*. The down-stream signalling cascade after TLR activation involves nuclear factor (NF)κB. Both *Nfkb1 *and *Nfkb2 *were significantly up-regulated at 4 h upon *E. coli *stimulation, but this was not seen after stimulation with *L. acidophilus*.

### Age dependent gut maturation does not influence early responses of epithelial cells towards Gram-positive and Gram-negative commensals

**Figure 5:**
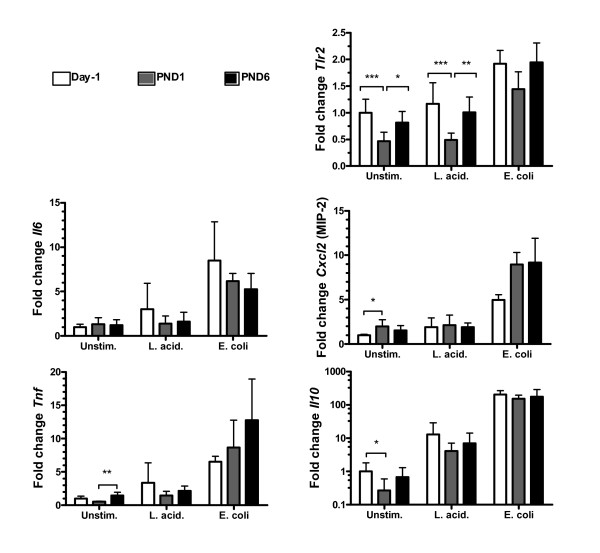
**Age dependent gut maturation does not influence early responses of epithelial cells towards G+ and G- commensals**. Epithelial cells from foetuses at Day-1 and neonates at post natal day (PND) 1 and PND6 from germfree mothers stimulated for 2 h with *L. acidophilus *NCFM or *E. coli *Nissle. Bars indicate mean and SD of 4 independent experiments with cells pooled from 6-10 pups (Day-1) or 2-3 pups (PND1 and PND6). ***p < 0.001, **p < 0.01, *p < 0.05. Data were normalised to *Actb *and then to the average of "Day -1 unstimulated" from the 4 experiments, which was defined to 1.

To unravel how age influences the IEC responsiveness independently of the microbiota we studied expression of 5 selected genes after *in vitro *stimulation with *L. acidophilus *and *E. coli *in primary IEC isolated from germfree mice at Day-1, post-natal day (PND)1 and PND6 (Figure 5). By keeping the mice germfree the only fluctuations in gene expression observed would be an effect of immune maturation with age. Interestingly, a significant transient drop in expression of *Tlr2 *was observed at PND1 in unstimulated IEC and *L. acidophilus *stimulated IEC. This could be a mechanism that allows the G^+ ^microbiota to establish at birth. The lower *Tlr2 *expression was accompanied by a decreased expression of *Il10 *and *Tnf *and an increased expression of *Cxcl2 *on PND1 in unstimulated cells. However, age did not significantly influence the response towards *L. acidophilus *and *E. coli*.

## Discussion

We here demonstrate that *L. acidophilus *NCFM and *E. coli *Nissle potently induce pro-inflammatory genes, a number of tolerance related genes, as well as genes involved in recruitment of immune cells to the GI tract. Although G^- ^bacterial stimulation impacts faster than G^+ ^bacteria, G^+ ^bacterial stimulation elicits a more sustained response giving rise to higher production of cytokines and chemokines. Moreover, *E. coli *induces transcription of *Tlr2*, a receptor for many G^+ ^bacteria, and down-regulates transcription of the LPS receptor, TLR4. *Tlr2 *expression was reduced immediately after birth independently of stimulation, but responses to stimulation were similar in IEC isolated from germ-free mice before and after birth.

Upon *E. coli *stimulation, foetal IEC expressed genes encoding the anti-inflammatory cytokines IL-10 and TSLP, and the chemokines KC, MIP-2 and MCP-1 potently and rapidly when compared to *L. acidophilus *stimulation. Relating the transcription data to the amount of protein measured in 18 h culture supernatant suggests a fast and transient up-regulation of cytokine and chemokine production induced by *E. coli *compared to a slower and more sustained up-regulation induced by *L. acidophilus*. This could be interpreted as fast induction of endotoxin tolerance during the culture period by *E. coli*. In line with our earlier studies in Caco2 cells [21], the two distinct bacteria differentially affected *Tslp *expression: *E. coli *enhanced *Tslp *expression, whereas *L. acidophilus *slightly reduced it. Production of TSLP by IEC is pivotal in maintaining gut homeostasis and indices on lower expression of *Tslp *in primary IEC from Crohn's disease patients than in healthy individuals have been reported [20]. The first microbial stimuli in neonate life probably affect TSLP production, which impacts on DC in the gut and thereby immune homeostasis. Pro- and anti-inflammatory gene expression as well as *Tlr4 *downregulation induced by *E. coli *may be indispensable for balancing the immune system in the gut and, as LPS exhibited similar effects, other G^- ^bacteria may have the same role.

Epithelial cell lines have been shown to develop a cross hypo-responsive phenotype after exposure to TLR2 and TLR4 ligands, probably due to altered signalling through TLR2 and TLR4 [10]. However, no studies have reported how these PRRs are regulated in primary IEC from a sterile gut upon first microbial encounters. We found that *E. coli *induced up-regulation of *Tlr2 *and down-regulation of *Tlr4*. It could be postulated that if the pioneer bacteria colonizing the sterile gut is a G^-^strain, LPS will down-regulate expression of TLR4 to enable LPS tolerance to be established. However, at 4 h the down-regulation seen at 2 h already approached the basal expression level (non-stimulated). This points towards a transient down-regulation of TLR4 by G^- ^bacteria-derived MAMPs, underlining that the expression of PRRs is tightly regulated in a dynamic fashion in order to initiate a quick response. As expression of *Tlr2 *was induced by *E. coli*, G^- ^commensals may induce cross hyper-responsiveness towards G^+ ^commensals in naïve primary IEC, which has not been reported before. Interestingly, *Clec7a *was down-regulated by *E. coli*, also pointing towards a yet undescribed cross-regulation of responsiveness to intestinal microorganisms. The induction of tolerance towards both G^+ ^and G^- ^MAMPs has been shown to depend on up-regulation of Tollip, which results in reduced phosphorylation of IL-1 receptor associated kinase (IRAK) and hence reduced NF-κB activation in both primary and immortalized IEC [10,25]. We measured expression of the genes encoding NFκB1, NFκB2, IKKβ (the IκB kinase), Tollip, and IRAK1. However, only *E. coli *induced up-regulation of *Nfkb1 *and *Nfkb2 *while expression of the other signalling proteins was not modulated by *in vitro *stimulation. Regulation of signalling relies on phosphorylation of the gene products, protein-protein interactions and protein translocation. Hence, transcriptional regulation is presumably more relevant for the responder cytokine and chemokine genes reported here.

During the first days of life, IEC develop and mature and crypts are formed. It has been demonstrated that germfree rats have impaired formation of crypt cells suggesting that the microbiota supports IEC growth and maturation [26]. *E. coli *Nissle, but not LPS, up-regulated *Tlr2 *expression, which may indicate a role for commensals in establishing intestinal integrity [27]. The fact that *E. coli *Nissle changed the expression of more genes and acted more potently than *E. coli*-derived LPS reflects that intact *E. coli *does not, as opposed to LPS, exclusively signal through TLR4. Lotz et al. [19] report higher secretion of MIP-2 and KC upon *in vitro *LPS stimulation at day -1 before birth compared with PND1 and PND6 in mice harbouring a conventional microbiota due to tolerance acquisition at birth. However, their study does not take into account that age might influence IEC maturation stage. In order to evaluate how IEC develop with age we studied the IEC responsiveness in germfree pups at Day-1, PND1 and PND6. Except for a transient drop in *Tlr2 *expression at PND1, we did not find strong age-dependent differences in the IEC response. The drop in *Tlr2 *expression at PND1 may allow G^+ ^commensals to colonise the gut without concomitant danger signals. Based on these findings, foetal IEC isolated from conventional mice represent an attractive supplement to polarised IEC cell line models for comparison of commensal bacteria as they are naïve to stimulation while being physiologically immature IEC and susceptible to tolerance induction. However, as IEC responses are clearly dose-dependent, *in vivo *experiments are still required to reveal the extent of contact between IEC and bacteria or bacterial components.

## Conclusions

Overall, our data confirm the hypothesis that the concomitant induction of chemokines, pro- and anti-inflammatory cytokines in enterocytes by the first-coming bacteria is indeed genus dependent. We conclude that *E. coli *and LPS may induce LPS-tolerance partly through very rapid and potent up-regulation of chemokine and cytokine genes and down-regulation of *Tlr4*, whereas stimulation by *L. acidophilus *Gram-positive commensals may be potentiated by the up-regulation of *Tlr2 *by Gram-negative bacteria.

## Methods

### Preparation of UV-killed bacteria

*L. acidophilus *NCFM was grown anaerobically in de Man, Rogosa, and Sharpe broth (Merck, Darmstadt, Germany) and *E. coli *Nissle aerobically in Luria-Bertani broth (Merck) overnight at 37 C. The cultures were harvested, washed twice in sterile phosphate-buffered saline (PBS) (Lonza, Basel, Switzerland) and re-suspended in 1/10 the growth volume of PBS. The bacteria used for *in vitro *stimulation were killed by a 40-min exposure to UV-light and stored at -80 C, as we from earlier studies have concluded that live and UV-killed bacteria elicit similar responses in epithelial cell lines [21]. Concentration was determined by lyophilisation. Endotoxin levels in *L. acidophilus *NCFM preparations were determined with the Pyrochrome kit (Ass. of Cape Cod, East Falmouth, MA, USA) to < 0.10 EU/ml in the highest concentration of stimuli used in cell culture experiments.

### Animal experiments

Conventional and germfree Swiss Webster mice were purchased from Taconic (Lille Skensved, Denmark), and housed under either specific pathogen-free conditions or in germfree isolators (as previously described [28]). Absence of colonising bacteria in germfree mice was confirmed by cultivation of faecal samples. Foetal IEC were isolated from foetuses derived from 4 conventional mothers. Caesarean section was performed on full-term pregnant females at gestation day 19 (referred to as Day -1), foetuses were killed immediately, and subsequently the small intestine was removed. Cells were pooled from 6-10 foetuses. Small intestinal tissue of neonatal mice was obtained from spontaneously delivered pups from germfree mothers at PND1 and PND6. Cells were pooled from 2-3 pups.

### Isolation of primary epithelial cells

The small intestine was placed in Hanks buffered saline (HBSS, Lonza) and cut into small pieces. The epithelial cells were detached from the underlying tissue by incubation in fresh HBSS containing 2mM EDTA at 37°C for 10 minutes with vigorously shaking every 3 minutes. Residual tissue was removed by passing the suspension through a 70μm filter. Cells were subsequently washed in cold PBS and re-suspended in culture medium (RPMI 1640 supplemented with 100 U/ml penicillin, 100μg/ml streptomycin, 2 mM L-glutamine, 10% (v/v) heat-inactivated FCS, all from Lonza). Cells were seeded in 48-well tissue culture plates (Nunc, Roskilde, Denmark) at 4x10^5^cells/500 μl/well. Fifty μl/well of bacteria or LPS O26:B6 (Sigma-Aldrich, St. Louis, MO, USA) were then added to obtain final concentrations of 1, 10, 30 or 100 μg/ml as indicated. The stimulus concentrations were chosen based on optimization experiments showing that smaller amounts of *E. coli *Nissle than *L. acidophilus *NCFM were required for stimulation of IEC. The cells were incubated for 2 h, 4 h or 18 h at 37°C in 5% CO_2 _and subsequently harvested by centrifugation and frozen in RNAlater (Qiagen, Hilden, Germany). The purity of the IEC was checked by staining for the lymphocyte marker CD45 (PE-labelled rat anti-mouse CD45 purchased from Abcam, Cambridge, UK) by flow cytometry. IEC contained 0.8 ± 0.4% CD45^+ ^cells at PND6 (staining with a matched isotype antibody (IgG2b) subtracted). Viability and cell numbers of IEC were determined by staining the cell nuclei with propidium iodide before and after cell lysis (reagents from Chemometec, Allerød, Denmark) and analysed with NucleoCounter (Chemometec). Viability of the IEC was evaluated during culture. We found that 23.5 ± 5.0% of freshly purified IEC were dead, 45.3 ± 2.9% after 2 h, 65.7 ± 6.5% after 4 h, 62.3 ± 6.6% after 7 h and 73.3 ± 7.6% after 24 h of culture.

### RNA purification and amplification

Samples were spun at 3000 *g*, 5 min, 4°C to remove RNAlater. RNA was extracted from the cell pellet using Mini Kit from Qiagen following the supplier's protocol for animal cells. The quantity and purity of extracted RNA was evaluated by Nanodrop spectroscopy (Thermo Scientific, Wilmington, DE, USA). cDNA was produced from app. 500 ng total RNA using High-Capacity cDNA Reverse Transcriptase Kit (Applied Biosystems, Foster City, CA, USA) according to the instructions of the manufacturer.

### Gene expression analysis by real-time polymerase chain reaction

For quantitative real-time polymerase chain reaction (RT-PCR), TaqMan Arrays (384-well Micro Fluidic Cards) were designed with the 20 TaqMan Gene Expression Assays (Applied Biosystems) listed in Table 1, permitting 8 randomized samples tested in duplicates on each card. The genes studied were chosen based on experiments comparing expression of more than 90 immune-related genes in intestinal cells of germfree and conventionally colonized mice (unpublished data). Genes with changed expression were included in the present study, as they were suspected to be affected by bacterial stimuli. To each cDNA sample (50 ng RNA in 50 μl) was added 50 μl TaqMan Universal PCR Master Mix (Applied Biosystems). Samples were mixed and loaded on the cards, which were centrifuged at 300 *g*, 1 min, 4°C and sealed. The PCR amplification was performed in standard mode using 7900HT Fast Real-time PCR system (Applied Biosystems). Additionally, single gene expression of *Cxcl2*, *Tnf*, *Il10*, *Il6 *and *Actb *was analysed (TaqMan Gene Expression Assays listed in Table 1). For each sample, 2 μl cDNA (3 ng/μl) was amplified in duplicates under universal fast thermal cycling parameters (Applied Biosystems) using TaqMan Fast Universal PCR Master Mix (Applied Biosystems) in a total reaction volume of 10 μl. Relative quantification (fold increase) was calculated by the comparative C_T _method. Briefly, C_T _is the threshold cycle, which is the cycle number where the amplified target reaches the defined threshold. The expression is normalised to the expression of a reference gene [C_T _= C_T_(target)-C_T_(reference)]. We evaluated *Actb *and 18S rRNA, which gave comparable results and chose to use *Actb *as reference gene. The efficiency of the PCR assays was tested by serial dilution of samples for 12 of the 24 genes on the TLDA arrays and was close to 100% (curve slopes between 3.3 and 3.4). Amplification specificity was similar for reference and target genes. The specificity of the assays was ensured by choosing intron-spanning TaqMan probes. In each dataset, a specific group of samples was used as calibrator (indicated in figure legends). Comparative gene expression was calculated as [C_T _= C_T_(target)- C_T_(calibrator))] and fold change (2^- CT^) values were plotted. Since C_T_(calibrator) = 0, Fold change = 1 for the calibrator group.

**Table 1:** Genes measured by Taqman low density array and (if bold also) by Taqman gene expression assays

Gene	Gene name	Protein	Assay ID
House keeping genes			
***Actb***	**actin, beta, cytoplasmic**	β-Actin	**Mm00607939_s1**
*18S*	eukaryotic 18S rRNA	-	Hs99999901_s1
Chemokines			
*Ccl2*	chemokine (C-C motif) ligand 2	MCP-1	Mm00441242_m1
*Ccl3*	chemokine (C-C motif) ligand 3	MIP-1α	Mm00441258_m1
*Cxcl1*	chemokine (C-X-C motif) ligand 1	KC	Mm00433859_m1
***Cxcl2***	**chemokine (C-X-C motif) ligand 2**	**MIP-2**	**Mm00436450_m1**
Cytokines			
***Il10***	**interleukin 10**	**IL-10**	**Mm00439616_m1**
***Il6***	**interleukin 6**	**IL-6**	**Mm00446190_m1**
*Tgfb1*	transforming growth factor, beta 1	TGF-β	Mm03024053_m1
***Tnf***	**tumor necrosis factor**	**TNF-α**	**Mm00443258_m1**
*Tslp*	thymic stromal lymphopoietin	TSLP	Mm00498739_m1
Regulation			
*Nfkb1*	nuclear factor of kappa light chain gene enhancer in B-cells 1	NFκB1	Mm00476361_m1
*Nfkb2*	nuclear factor of kappa light chain gene enhancer in B-cells 2	NFκB2	Mm00479807_m1
*Ikbkb*	inhibitor of kappaB kinase beta	IKKβ	Mm00833995_m1
*Irak1*	interleukin-1 receptor-associated kinase 1	IRAK1	Mm00434254_m1
*Tollip*	toll interacting protein	Tollip	Mm00445841_m1
Pattern recognition receptors			
*Clec7a*	c-type lectin domain family 7, member a	Dectin-1	Mm00490960_m1
***Tlr2***	**toll-like receptor 2**	**TLR2**	**Mm00442346_m1**
*Tlr4*	toll-like receptor 4	TLR4	Mm00445274_m1
*Ly96*	lymphocyte antigen 96	MD-2	Mm00444223_m1

### Cytokine quantification in culture supernatants

The production of MIP-2, IL-10, -6, TNF-α was analysed using commercially available ELISA kits (R & D systems, Minneapolis, MN, USA).

### Statistical analysis

GraphPad Prism version 4.03 (GraphPad Software, San Diego, CA, USA) was used to perform two-way ANOVA with Bonferroni post-test. Although fold change is plotted in gene expression experiments, statistical analysis was performed on C_T_values as these are assumed normally distributed as opposed to the fold change values.

## Authors' contributions

LHZ participated in the study, performed data analysis and wrote the manuscript. LNF and SBM designed and performed the study, carried out statistical analyses and edited the manuscript. MBK, TRL and CN participated in cell experiments and expression studies. HF participated in the design of the study and edited the manuscript. All authors read and approved the final manuscript.
